# A Postoperative Pain Management Mobile App (Panda) for Children at Home After Discharge: Usability and Feasibility

**DOI:** 10.2196/12305

**Published:** 2019-07-04

**Authors:** Dustin Dunsmuir, Helen Wu, Terri Sun, Nicholas C West, Gillian R Lauder, Matthias Görges, J Mark Ansermino

**Affiliations:** 1 Department of Anesthesiology, Pharmacology & Therapeutics University of British Columbia Vancouver, BC Canada; 2 Department of Pharmacology and Therapeutics McGill University Montreal, QC Canada; 3 Department of Pediatric Anesthesia BC Children’s Hospital Vancouver, BC Canada; 4 BC Children’s Hospital Research Institute Vancouver, BC Canada

**Keywords:** pain management, pain, postoperative period, outpatients, mobile apps, child, parents

## Abstract

**Background:**

Emphasis on outpatient pediatric surgical procedures places the burden of responsibility for postoperative pain management on parents or guardians. Panda is a mobile phone app that provides scheduled medication alerts and allows parents to track their child’s pain and medication administration. We have previously tested and optimized the usability and feasibility of Panda within the hospital setting.

**Objective:**

The purpose of this study was to evaluate and optimize the usability and feasibility of Panda for use at home based on alert response adherence (response to any medication notification within 1 hour) and parents’ satisfaction.

**Methods:**

Parents or guardians of children aged 3 to 18 years undergoing day surgery were recruited to use Panda at home for 1 to 7 days to manage their scheduled medications and to assess their child’s pain. After the surgical procedure, a research assistant guided parents through app setup before independent use at home. We aimed to recruit 10 child-caregiver pairs in each of three rounds of evaluation. Each user’s adherence with the recommended medication alerts was analyzed through audit-trail data generated during the use of the app. We used the Computer System Usability Questionnaire and a poststudy phone interview to evaluate the app’s ease of use and identify major barriers to adoption. Suggestions provided during the interviews were used to improve the app between each round.

**Results:**

Twenty-nine child-caregiver pairs participated in three rounds, using the app for 1 to 5 days. Alert response adherence (response to any medication notification within 1 hour) improved as the study progressed: participants responded to a median 30% (interquartile range [IQR] 22%-33%) of alerts within 1 hour in round 1, and subsequently to median 60% (IQR 44%-64%) in round 2 and median 64% (IQR 56%-72%) in round 3 (*P*=.005). Similarly, response times decreased from median 131 (IQR 77-158) minutes in round 1 to median 31 (IQR 18-61) minutes in round 2 and median 10 (IQR 2-14) minutes in round 3 (*P*=.002). Analysis of interview feedback from the first two rounds revealed usability issues, such as complaints of too many pages and trouble hearing app alerts, which were addressed to streamline app function, as well as improve visual appearance and audible alerts.

**Conclusions:**

It is feasible for parents or guardians to use Panda at home to manage their child’s medication schedule and track their pain. Simple modifications to the app’s alert sounds and user interface improved response times.

## Introduction

### Background

The number of pediatric outpatient surgical procedures is increasing [1,2]. A range of medications may be prescribed to manage pain during recovery from ambulatory procedures [3]. Unfortunately, studies have suggested that children frequently experience significant pain following discharge from hospital [4,5] and that poorly managed postsurgical pain can contribute to significant long-term problems [6,7]. Pain may be managed less effectively by parents or caregivers at home than by health care professionals in hospital due to the challenges faced in assessing a child’s pain, following prescription schedules, and calculating doses. There can also be misconceptions about the effects and safety of medications [5,8,9].

There are opportunities for mobile phone apps to guide and improve postoperative pain management for patients after they leave the hospital. Thus far, apps used for self-management of acute sickle cell crisis [10], chronic pediatric cancer pain [11], burn recovery [12], and diabetes [13] have shown promise with patient engagement [10], reduction of anxiety or pain scores [12], improved self-management [13], and overall user satisfaction. A recent review of commercially available mobile phone apps for postoperative pain management demonstrated a lack of evidence-based content [14], and few were designed for use in children. The PediPain app [15] developed by SickKids (a major pediatric hospital in Toronto, ON, Canada) is a pediatric pain and medication dosing app, but it is designed for nurses and doctors in the hospital. Thus, there is still a need for an app to support parents in managing their child’s pain after they leave the hospital.

To address this, the Digital Health Innovation Lab (DHIL) at BC Children’s Hospital (BCCH, Vancouver, BC, Canada) has developed Panda, a mobile phone app designed to support parents in performing three primary tasks: (1) assessing their child’s pain using digitized versions of validated self-report pain tools, (2) scheduling medication reminders using alerts, and (3) tracking medications administered and pain histories. The app has been previously evaluated for safety and ease of use and was improved following several rounds of usability and feasibility testing in hospital [16]. In the feasibility portion, parents used the app while their child was admitted to hospital following surgery. This allowed us to evaluate the app’s usability and feasibility within a controlled environment. The final round of testing demonstrated marked improvement, with 84% (31/37) of app alerts being responded to by parents within 1 hour, and 93% (27/29) of parents reporting that the app was easy to use [16].

The Panda app contains four pediatric self-report pain scales, including established pediatric pain scales, the Faces Pain Scale Revised [17] (ages 4 years and older) and Color Analog Scale [18] (ages 5 years and older), for which we previously demonstrated good agreement between new digital mobile phone versions and the original paper or plastic versions [19]. Additionally, two simplified pain scales, the Simplified Faces Pain Scale and Simplified Concrete Ordinal Scale, have also been designed for mobile phone use and validated in younger children aged 3 to 5 years [20]. Panda was primarily made for parental use, with the exception of the self-reported pain scales, which required the child (aged 3-18 years) to interact with the app to indicate their level of pain.

### Objectives

The primary objective of this study was to demonstrate the feasibility of the Panda app with parents or guardians at home; specifically, will parents regularly use the app and consider it a useful aid for administering pain medications to their child? The measure of feasibility was assessed by the degree of alert response adherence (responding to any medication notification within 1 hour) and parental-reported satisfaction with the app during the postoperative care of their child. The secondary objective was to improve the app iteratively by identifying usability issues that arose at home. Usability issues were any undesired or confusing aspects of the app (eg, the appearance or page progression) that caused interactions or behavior that led to undesired or unexpected outcomes, ultimately reducing use of the app and thus negatively affecting feasibility.

## Methods

### Study Design

This was an observational study to investigate the feasibility of parents using Panda at home to manage their child’s pain after an outpatient surgical procedure. Parents were sent home with the Panda mobile phone app; their use of the app was logged by an automated audit function (recording of all buttons pressed, text entered, and other interactions with the app as time-stamped entries in a log file). Poststudy telephone interviews (Multimedia Appendix 1) were conducted with the participants (parents), and an online questionnaire about the usability of the app was used to collect feedback from each participant. This quantitative usage data and qualitative feedback were then analyzed for common problems and themes and used to improve the app.

Approval from the University of British Columbia-Children’s and Women’s Health Centre of BC Research Ethics Board (H17-00645) was obtained. The study was conducted between May and August 2017 at BCCH in Vancouver, BC, Canada.

### Participants

The inclusion criteria for the participants of the study were parent or guardian aged 18 years or older, caring for a child aged 3 to 18 years undergoing an elective ambulatory surgical procedure for which there was minor anticipated postoperative pain. The attending anesthesiologist informed the research assistant (HW) of procedures associated with postoperative pain. The child must have been discharged from hospital on the day of their surgery and have planned postdischarge analgesic medications, including nonsteroidal anti-inflammatory drugs, acetaminophen, or opioids, for at least two days. Parents or guardians or children with hearing or visual impairment, neurologic injury or psychomotor dysfunction, developmental delay, cognitive neuromuscular inability to operate the app, or inability to follow study instructions in English were excluded. Eligible participants were identified on the operating room schedule by a study research assistant before being approached. Written, informed parental consent and, if appropriate, child assent, were obtained in the surgical day care unit before the child’s operation.

### The Panda App

**Figure 1:**
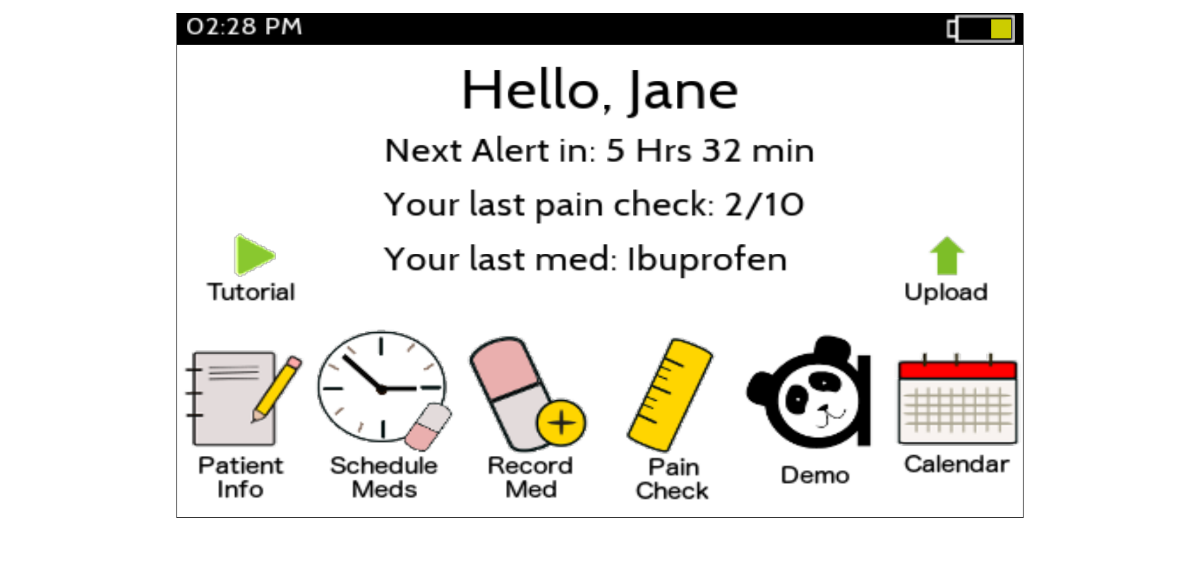
Home screen of Panda showing the available menu buttons.

The Panda app has been described previously [16]. The initial app setup includes selecting the appropriate pain scale of the Color Analog Scale, Faces Pain Scale Revised, Simplified Faces Pain Scale, or Simplified Concrete Ordinal Scale (with a recommendation made automatically based on age) and entering a medication schedule according to a child’s prescribed medication. The user has the option of disabling the medication and pain assessment alarms at inconvenient times, such as when they would be sleeping. Then, at the regularly scheduled medication administration times, the Panda app alerts the user via a pop-up notification on their mobile phone. On opening the app, a notification displays the name(s) of the medication(s) to give and offers three response options: proceed, snooze, or skip. Selecting *proceed* directs the user to the standardized pain scale, and then guides them through medication safety checks before confirming the medication(s) being given to their child. *Snooze* allows the parent to delay the reminder. Finally, there was an opportunity to *skip* giving the medication. Additional functions of the app (Figure 1) include editing the patient information and medication schedule, off-schedule medications, off-schedule pain checks, a calendar showing past medication and pain, and a demo mode allowing the user to practice the response to a medication alert.

### Procedures

After written informed consent, the participant completed a prestudy questionnaire about their general knowledge and use of mobile phone apps. For the study, each enrolled participant had the choice of using his or her personal Android or iPhone device, or an iPod Touch provided by DHIL. A research assistant guided the participant in downloading the app and provided an orientation of the Panda app’s primary functions during a suitable time during their hospital stay. The research assistant also guided the participant through the setup of the app by entering their child’s demographics, operation information, and medication schedule as prescribed by the physician after the operation. The research assistant also ensured that the participants were satisfied with the default selected pain scale, which could be changed if requested. Panda includes a tutorial video directly within the app to help the user remember how to use all its functions and features. In addition, each pain scale contains a training page and optional audio instructions for the child.

At home, the participant was expected to use the Panda app for guidance with their child’s pain assessment and management of prescribed analgesic medications. All actions within the Panda app were logged within the audit trail. App data were uploaded to a Research Electronic Data Capture (REDCap) database [21], hosted at the BCCH Research Institute. This enabled the research team to track each participant’s usage of Panda daily during the study period.

The observation period lasted for 1 to 7 days. A research assistant was available by phone, email, or text messaging to answer questions and resolve issues related to the function of the app during regular working hours (9 am - 5 pm, Monday - Friday). The app audit trail on REDCap enabled the research assistant to see when each participant stopped using the app. The research assistant called the participant for the telephone interview within 3 days of their use of Panda stopping completely. The research assistant performed a structured 12-item interview (Multimedia Appendix 1) with each participant about their general satisfaction with the app, specifically identifying any barriers to use, the utility of specific features, and general ease of use of the app.

A final upload of each participant’s usage data was performed before the app was deactivated and deleted from the participant’s personal device; in the case of a borrowed iPod Touch, it was mailed back to the research office using prepaid envelopes. Finally, participants were emailed an electronic Computer System Usability Questionnaire (CSUQ) [22] survey via REDCap, which consisted of 19 questions inquiring about general usability of the app, each on a 7-point Likert scale. Both the phone interview and the CSUQ measured participant satisfaction. However, the interview was designed specifically for Panda and provided the team with specific qualitative feedback, and the CSUQ provided standardized quantitative data for comparison of rounds. If the survey was not completed within 2 days, the survey email invitation was resent once. If it was still not completed, that participant’s data were analyzed without any CSUQ responses.

### Data Analysis

A pragmatic sample size of N=30 was chosen, which is typically suggested for usability evaluations and matches similar studies [11,16,23]. This study was designed using three rounds of data collection, each consisting of 10 participants. After each round, data were extracted from the audit log, including the timing and response (proceed, skip, or snooze) to all app notifications and any off-schedule medications recorded or pain checks done. For each participant, we calculated the duration of study participation, the median response time between each scheduled alert time and the user’s response time, the alert response adherence (response within 1 hour), the median pain scores reported, the proportion of pain checks in which a participant said there was “no pain” (responded to the alert, but specifically said there was no pain and therefore were not shown a pain scale; counted as a score 0), the number of doses of acetaminophen (the most common medication given), and the interval between these doses. These values were compiled across all participants in a round and then compared between rounds using the Kruskal-Wallis test for continuous data; a critical alpha of .05 was used to determine statistical significance. We considered a response to a medication reminder within 1 hour to be compliant with the medication schedule.

Usability was determined by the median CSUQ score, and participant satisfaction was assessed by analyzing the poststudy interview responses. Median values were calculated per question across all completed questionnaires, excluding response of “not applicable” or “N/A.” Responses to interview questions were collated and used to identify common themes [24]. This analysis was done after each round and used to target areas for improvement in app design before the next round. After each round, the software developer (DD) modified the Panda app based on participant feedback collected from the interview and the device-recorded audit of app usage.

## Results

### Participants

**Figure 2:**
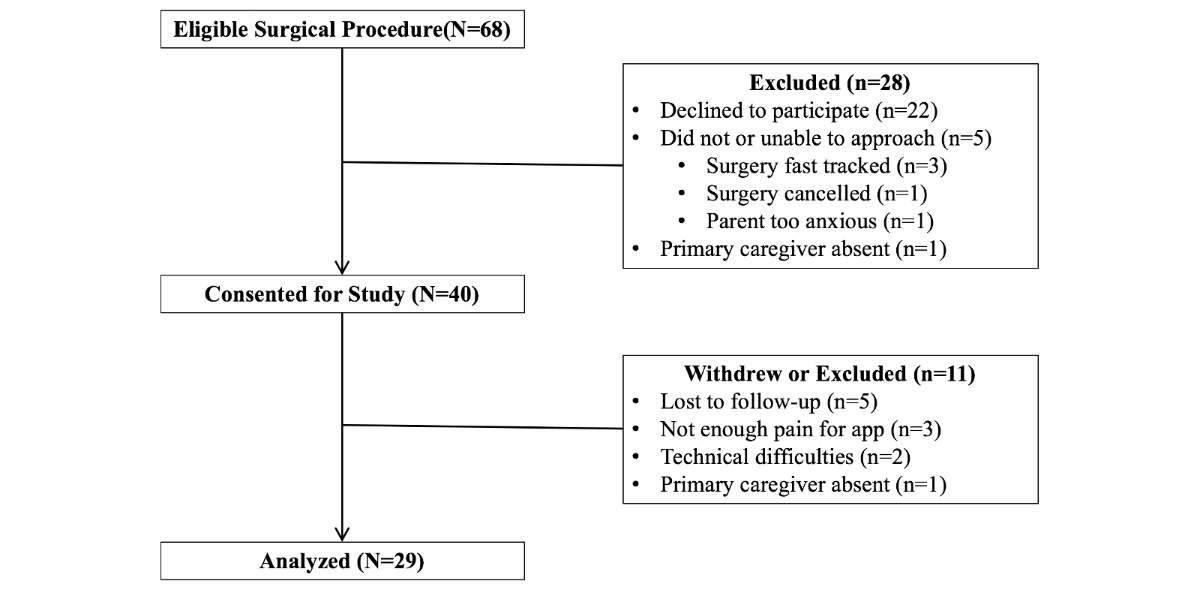
Study participant screening and enrollment.

Between May and August 2017, the research assistant identified 68 eligible participants; 40 participants were recruited, and 29 participants successfully completed all questions within the prestudy questionnaire, used the Panda app at home for at least one day (see Figure 2), and then took part in the poststudy telephone interview. Most of the 22 potential participants that declined did not give a reason, but reasons given included the expectation that their child would not be in enough pain to require the app (n=3), they would not require help from an app (n=3), or because they were anxious about their child’s procedure and did not wish to be distracted with participation in the research project (n=1).

Of the 29 participants, 3 were in their twenties, 11 were in their thirties, 11 were in their forties, and the remaining 4 were older than 50 years. The majority of participants (23/29, 79%) were female, and 90% (26/29) were daily mobile app users. Although they used apps regularly, Panda was a novel app to them as only 52% (15/29) had previously used some form of health or fitness app, and 45% (13/29) reported they would normally use their memory alone to remind themselves when to give medications. In addition, only 38% (11/29) would normally use any type of pain scale (such as numbers 1-10) to have their child communicate their degree of pain. The median age of the participating children was 8 years (interquartile range [IQR] 4-12), and there were 18 males and 11 females. Surgical procedure types included dental (n=6); orthopedic (n=7); general surgery (n=6); ear, nose, and throat (n=6); and plastic surgery (n=4).

### App Usage

**Figure 3:**
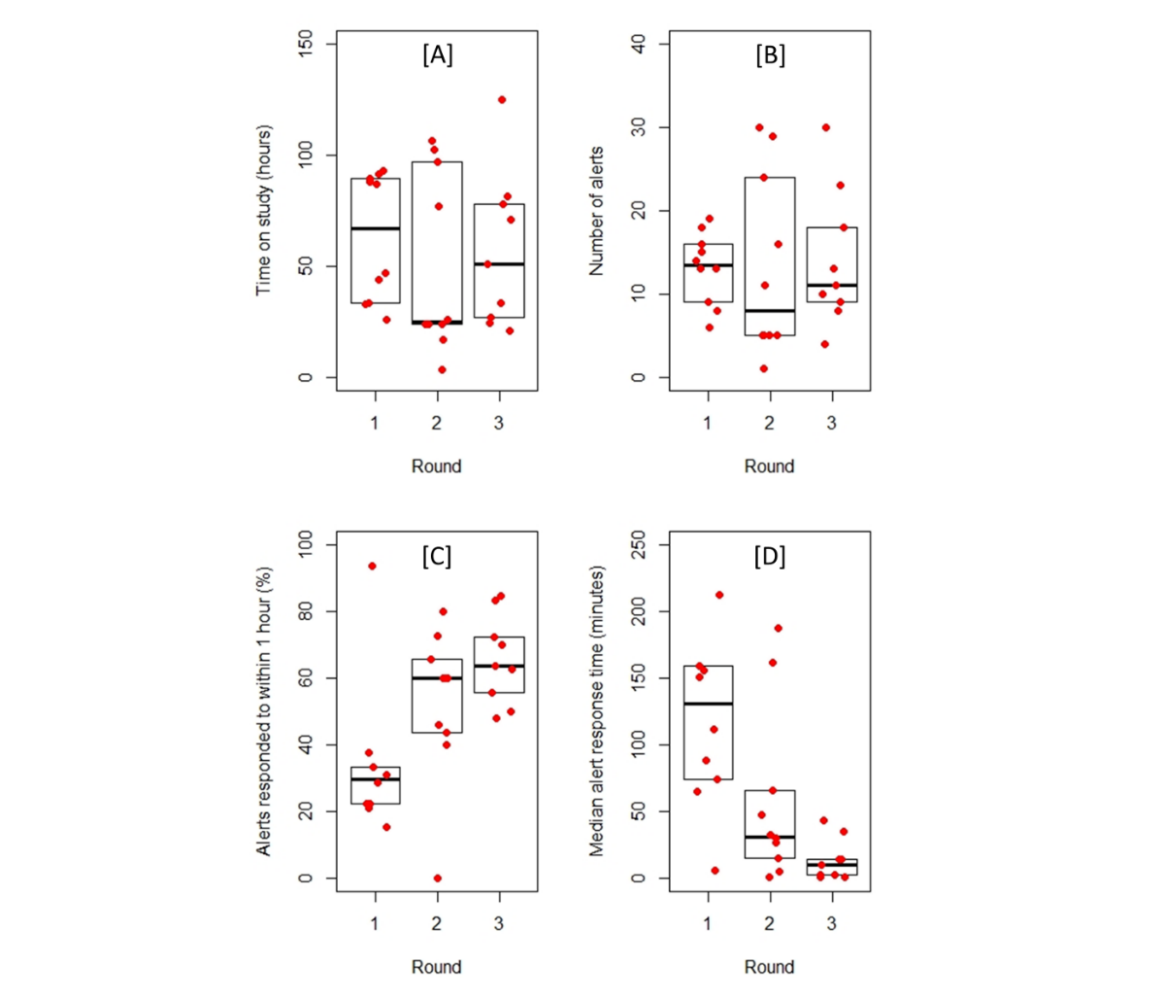
Changes in usage between rounds. Data are summarized as boxplots; the horizontal line in the middle of each box indicates the median, and the top and bottom borders mark the 25th and 75th percentiles, respectively. Individual data are overlaid as red dots. (A) duration of study participation, (B) number of alerts during that time, (C) proportion of alert responses within 1 hour, and (D) median response times by round, in which one outlier has been removed (in round 1, one participant had a median response time of 662 minutes).

Participants used Panda for a maximum of 5 days during their child’s postoperative recovery at home. Five participants used the DHIL-provided iPod Touch device, four participants used their own Android phones, and the other 20 used their own iPhones. Usage of the app during the study was variable, with some participants reporting that their child was not in enough pain for the app to be needed for very long. The full usage log was obtained from all 29 participants. The duration of app usage varied from only one alert the morning after surgery (one participant in round 2) to 30 alerts over 4 or 5 days (one participant each in rounds 2 and 3). There was no significant difference between the rounds in terms of duration of study participation, median pain scores, proportion of pain scores recorded as “no pain,” number of doses of acetaminophen given, or the interval between these doses (Table 1). Response to alerts improved from one round to the next: participants responded to more alerts within 1 hour (*P*=.005) and median response times decreased significantly (*P*=.002; Table 1 and Figure 3).

Most children reported no pain (score 0/10), low pain (score 1-3/10), or moderate pain (score 4-7/10; see Table 1). However, 6 of 29 (21%) children reported severe pain (score 8-10) at some point during the study, and 4 of 29 (14%) reported severe pain repeatedly (ie, more than three times). All four of these children were given acetaminophen and ibuprofen, and two of them received an additional analgesic medication, such as oral morphine. The median interval between doses of acetaminophen for these four children were 6 hours, 10 hours, 11 hours, and 19 hours, respectively.

**Table 1:** Summary of response times, pain scores, and administration of acetaminophen (N=29).

Measurements	Round, median (interquartile range)			*P* value^a^
	Round 1 (n=10)	Round 2 (n=10)	Round 3 (n=9)	
Time on study (hours)	67 (36-89)	25 (24-92)	51 (27-78)	.39
Responded to alert before next alert (%)	65 (48-82)	81 (70-100)	89 (75-90)	.07
Responded to alert within 1 hour (%)	30 (22-33)	60 (44-64)	64 (56-72)	.005
Median response time (minutes)	131 (77-158)	31 (18-61)	10 (2-14)	.002
Number of pain scores recorded	8 (6-13)	8.5 (4-17)	8 (6-12)	>.99
Median pain score	0 (0-2.5)	3 (0.3-5.6)	0.8 (0-2.5)	.26
Proportion of pain scores recorded as “no pain” (%)	66 (39-84)	22 (2-58)	20 (17-80)	.10
Doses of acetaminophen given	4 (2-8)	4.5 (1-7)	5 (2-6)	.92
Median interval between acetaminophen doses (hours)^b^	9.3 (4.8-9.9)	11.1 (8.9-13.1)	8.8 (7.1-10.2)	.24

^a^*P* values calculated using the two-tailed Kruskal-Wallis test.

^b^Prescriptions for acetaminophen varied from every 4 hours (14 participants) to up to every 12 hours (1 participant).

### Participant Feedback

After the study, 22 of 29 participants (76%) completed the CSUQ. Across three rounds, the rating was median 2 (IQR 1-3) or “agree.” The most positively rated statement was “It was easy to learn using this interface” with a rating of median 1.5 (IQR 1-2). The structured poststudy telephone interview helped identify barriers to using the app. When specifically asked “Was responding to a medication alert clear to you?” 28 of 29 (97%) participants responded positively. After a thematic analysis of participant responses, several issues were identified (Table 2). The first three issues in Table 2 were addressed through changes to the app as described subsequently. As in our earlier study, some issues occurred throughout all three rounds of the study and were not addressed due to conflicting with the purpose of the app (Table 2). For example, one participant stated:

The main thing is having the medications shift once you give an off-schedule medication.

Another commented:

Would prefer faces that children are more familiar with, like emojis. Include a number scale because my son is used to expressing pain on a scale from 1-10.

There were additional feature requests that were beyond the scope of the app, such as including medication doses and allowing use from two different devices (both parents managing the child’s pain collaboratively).

**Table 2:** Common feedback from participants by study round (N=29).

Feedback	Round 1 (n=10), n	Round 2 (n=10), n	Round 3 (n=9), n
Too many app screens during medication safety check	3	1	0
Trouble hearing alerts	4	1	0
App graphics not visually appealing (too “immature”)	3	3	0
Wanted a more flexible schedule for medications	3	1	4
Did not want child to use the pediatric pain scales	4	2	0

**Figure 4:**
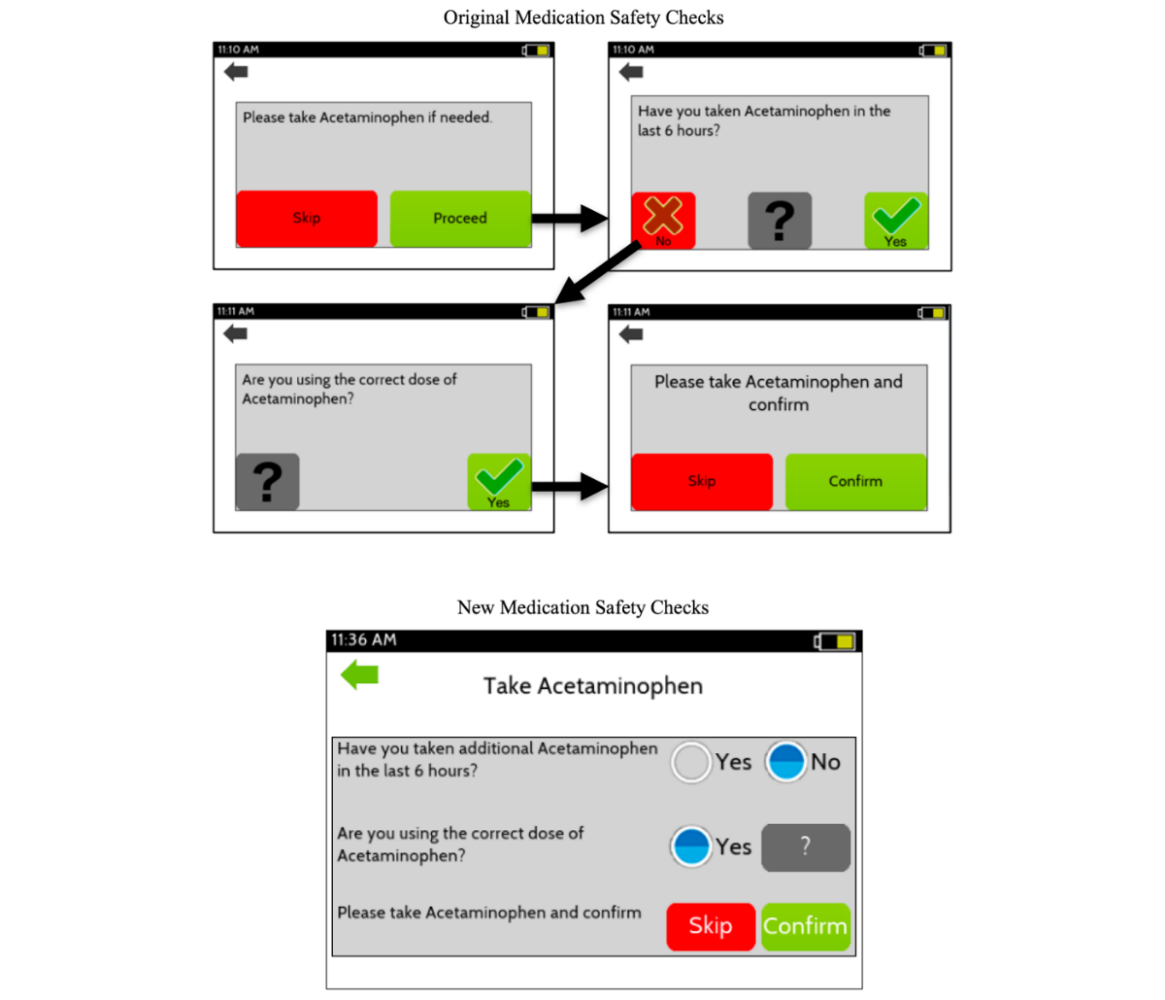
The changes made to the medication safety checks after round 1.

### App Changes

After the first round, changes were made to reduce the number of pages by combining all medication safety checks into a single page (Figure 4). To address the audibility of alerts, an alert “test” button was added to the initial app setup that triggered an alert 3 seconds later to ensure alerts were both audible and visible on their personal phone. After the second round, the alert sound was changed to a more obvious alert tone, the height and weight were made optional within the demographics, and the home page icons were also redesigned to address the user criticism (Figure 1).

## Discussion

### Principal Findings

This study evaluated the Panda app in a real-world setting, with parents or guardians successfully using it for their child’s postdischarge pain management at home. There was a statistically significant decrease in the time to respond to the app between rounds of testing and an increased proportion of responses within 1 hour, indicating improved adherence to the app notifications. From interview feedback, key issues with the app were identified and the app was improved between rounds.

According to the prestudy questionnaire, only half of the participants were familiar with health or fitness apps, but the CSUQ results and poststudy interviews suggested that Panda was easy to learn and that the most common action, responding to an alert, was clear to the majority of participants. Common complaints about the app included the graphics and alert sounds, which can be attributed to preference and app aesthetics rather than the app usability and functionality. Nonetheless, we addressed these concerns in an effort to improve Panda’s adoption. Almost all the participants responded positively to Panda and were able to use it with minimal supervision; therefore, it can be said that Panda is feasible for use by parents or guardians at home.

The purpose of this study was to evaluate feasibility and to address outstanding usability issues that may not have been identified during our previous study in the hospital [16], rather than to track any clinical benefits that may be provided by the app. Nonetheless, we noted that 4 of 29 (14%) children who participated in the study did have severe pain that lasted for a significant period of time. There was a high degree of variability in dosing frequency of analgesic medications recorded within the app with the median time for three of these four children being much longer than the prescribed medication schedule despite persistent and severe pain. This may suggest that some parents were not following the clinical guidance provided by the app or that the child was experiencing severe pain despite regular medications; either scenario would warrant clinical attention and provide a window of opportunity for health care intervention to potentially change outcomes. Future development of in-app educational content specific to at-home pain management and two-way communication between the app user and the clinical team may help optimize pain management.

Several research groups are beginning to investigate the use of mobile phone apps to track and promote postoperative rehabilitation, including requirements for patient action within a prescribed recovery program. For example, Jaensson et al [25,26] developed Recovery Assessments by Phone Points (RAPP), a mobile phone app for monitoring and assessing postoperative recovery, and showed that the mobile app was more cost-effective than a control group receiving standard care [27]. Symer et al [28] developed an app to track recovery after abdominal surgical procedures that they suggest will be able to improve outcomes. A recent pilot study by Pecorelli et al [29] reported positive results with a tool to record patient adherence to care processes and patient-reported outcomes as part of an enhanced recovery program for bowel operations. Other researchers have developed an app to monitor wound recovery using mobile phone technology [30,31]. It has also been shown by Lu et al [32] that adherence with pre- and postoperative protocols for reducing surgical site infections can be increased using simple automated messaging systems. These examples illustrate the perceived potential for mobile phone technology in the perioperative period. There are potential advantages for patient outcomes, as well as possible cost-saving benefits for hospitals if these technologies can be shown to facilitate a safe alternative to postoperative care in hospital or reducing returns to hospital for follow-up issues such as pain and wound infection.

Mobile health apps provide an opportunity to optimize pediatric pain management at home [33]. In the context of postoperative care, it may be useful to include educational content on general care, procedure-specific guidance, suggestions for self-management of acute pain, information on prescribed and alternative medications, and instructions for what to do in case of patient deterioration, among other things. These components were beyond the scope of the Panda app, but some are being considered in future iterations ahead of full-scale implementation.

### Limitations

This study considered alert response adherence as a surrogate measure of medication adherence, but with the use of the app by parents independently at home there is no way to confirm if the medication was actually given. The app ensured that the participants were made aware of when their child’s medications were due; however, it was the participant’s (and patient’s) choice whether to comply or not.

The target primary user for Panda in this study was exclusively a parent or guardian. Despite the child age range including adolescents, we previously found teens to have a low task completion rate within Panda [16]. A pain management app for teens should be designed specifically for them, and any additional educational content would likely be presented quite differently than for parents or guardians. For example, Stinson et al [11] gamified pain management to motivate adolescents with cancer to use their app. Our study did not collect any feedback directly from the pediatric patients given their varying age but instead relied on feedback from the main app user, the parent. Feedback on the pain scales was previously collected in our testing of the digital versions of the scales [19].

The heterogeneity of the study cohort meant that some patients reported very little pain during their postoperative recovery. This may have biased their parents against using the app, which is hard to interpret from the data available. Future evaluation of the app may be targeted at more specific age and procedure groups when we aim to evaluate the impact of Panda on patient outcomes.

Although some users expressed that the app was easy to use, there were additional features suggested, which were not always aligned with the current goals of the app (eg, improving simplicity over ensuring medications were administered safely). The main goal of the Panda app is to help with management of postoperative pain by increasing adherence to the medication schedule prescribed or recommended by their health care providers. Panda allowed participants to determine the time of the first dose of medication and to turn off any alert (such as alerts while sleeping), but all alert times were predetermined according to the selected time interval (give medication every 4 hours, 6 hours, etc). Participants expressed a desire for more control of the schedule by being able to adjust the predetermined schedule to their convenience; however, allowing this would require an intelligent algorithm to ensure that the medication schedule remained in accordance with safe dosing. The addition of dynamic pain medication scheduling to the app may hinder the simplicity and predictability of the schedule.

### Future Work

We are planning to enhance Panda’s utility through the addition of two-way communication features before releasing it on the public Google and iOS app stores. Clinicians have expressed the usefulness of better follow-up information on their patients postoperatively. It is clear from some of the children’s pain scores that medication alerts alone may not be enough to eliminate severe pain during the recovery period, and intelligently communicating these pain scores to clinicians could identify specific children who might require additional interventions. Additionally, parents would benefit from a new and more convenient means of communication with the clinical team during their child’s postoperative recovery. Hence, we will be adding a two-way communication system to the Panda platform, allowing the clinicians access to a dashboard view of their patients’ pain scores and medication usage as sent from the Panda app.

### Conclusions

The Panda app is feasible to be used by parents at home for the management of their child’s postoperative pain. Apps such as Panda can take advantage of the ubiquity of mobile phones to provide a useful pain management tool in parents’ pockets. Overall, the Panda app can serve as a support system for parents to help manage their child’s pain after discharge from hospital, potentially preventing unnecessary pain and associated sequelae and the need to reaccess health care resources.

## Appendix

Multimedia Appendix 1 Telephone interview questions.

## Abbreviations

BCCH: BC Children’s Hospital
CSUQ: Computer System Usability Questionnaire
DHIL: Digital Health Innovation Lab
IQR: interquartile range
REDCap: Research Electronic Data Capture
