# Supplementary material for: A Postoperative Pain Management Mobile App (Panda) for Children at Home After Discharge: Usability and Feasibility
Source: JMIR Perioper Med. 2019 Jul 4;2(2):e12305. doi: 10.2196/12305 (PMC7709843; doi:10.2196/12305)
Supplement: Multimedia Appendix 1 [file periop_v2i2e12305_app1.docx]

______________________________________________________________________________

1. Generally, what did you think about using the app? (Easy or difficult?)
2. What did you like the least or find most difficult about using the app?
3. What did you like the most about using the app?
4. What would you add or change to improve the app? Do you think the app could do more?
   1. What do you think about the appearance or layout of the app and how could this be improved?
5. How do you feel about the set up procedure? Was it straightforward?

1. In terms of the medication alert pop-up which said: "Skip", "Snooze" and "Proceed"?
   1. What option do you think you chose most often?
   2. Was responding to a medication alert clear to you?
   3. What do you think about the alert sound?
2. When confirming a medication is taken the app asks “Have you given the medication in the last ‘X’ hours? Yes or No?” Did you understand this question?
3. Did you edit any medications or redo any pain scores from the calendar page and was it easy to do?
   1. Did you use the feature where you can add notes to a specific entry? Was it useful?
4. Did you record any off schedule medications? Was it easy to do?
5. Did you do pain checks only when alerts went off or did you do them off schedule as well? Was this easy to do?
6. Were you awakened by any alerts in the middle of the night. How do you feel about late night alerts?
7. Do you have any other feedback or anything else you want to say about the app?
